# Myths in Medicine and Old-Time Doctors

**Published:** 1885-05

**Authors:** 


**Myths in Medicine and Old-Time Doctors.** By ALFRED C. GARRATT, M. D., Fellow of the Massachusetts Medical Society. New York and London: G. P. Putnam's Sons. The Knickerbocker Press. 1884.

This work is addressed to the profession, especially the younger portion of it, and aims to record medical history and the successive schools and sects. The author divides the work into seven chapters, of which the first treats of eminent physicians in ancient times from Hippocrates to Galen; the second, the dark ages, Mohammedanism in Europe, medicine, Christianity, and law-blighted; the third, the medical profession about two hundred years ago; the fourth, old-time theory of the nature and cause of nervous maladies; the fifth, the treatment of nervous distempers, as then practiced; the sixth, what was alchemy in the seventeenth century? the seventh, analysis of homœopathy. An appendix finishes the volume, containing references and corroborating testimony on the question of homœopathy. Under these various heads, the author furnishes a variety of interesting material, presented in a very pleasant and easy style. The book will subserve a useful purpose to the general reader as well as to medical men. We have been much interested in its perusal and commend it to those who have leisure to scan its pages.

